# Using Stem Cells to Model Diseases of the Outer Retina

**DOI:** 10.1016/j.csbj.2015.05.001

**Published:** 2015-05-06

**Authors:** Camille Yvon, Conor M. Ramsden, Amelia Lane, Michael B. Powner, Lyndon da Cruz, Peter J. Coffey, Amanda-Jayne F. Carr

**Affiliations:** a The London Project to Cure Blindness, Division of ORBIT, Institute of Ophthalmology, University College London, 11-43 Bath Street, London EC1V 9EL, UK; b NIHR Biomedical Research Centre at Moorfields Eye Hospital NHS Foundation Trust, UCL Institute of Ophthalmology, London, EC1V 2PD, UK; c Center for Stem Cell Biology and Engineering, NRI, UC, Santa Barbara, USA

**Keywords:** Disease models, Induced pluripotent stem cells, Retinitis pigmentosa, Age related macular degeneration, Leber congenital amaurosis, Inherited retinopathy

## Abstract

Retinal degeneration arises from the loss of photoreceptors or retinal pigment epithelium (RPE). It is one of the leading causes of irreversible blindness worldwide with limited effective treatment options. Generation of induced pluripotent stem cell (IPSC)-derived retinal cells and tissues from individuals with retinal degeneration is a rapidly evolving technology that holds a great potential for its use in disease modelling. IPSCs provide an ideal platform to investigate normal and pathological retinogenesis, but also deliver a valuable source of retinal cell types for drug screening and cell therapy. In this review, we will provide some examples of the ways in which IPSCs have been used to model diseases of the outer retina including retinitis pigmentosa (RP), Usher syndrome (USH), Leber congenital amaurosis (LCA), gyrate atrophy (GA), juvenile neuronal ceroid lipofuscinosis (NCL), Best vitelliform macular dystrophy (BVMD) and age related macular degeneration (AMD).

## 1 Introduction

**Fig. 1:**
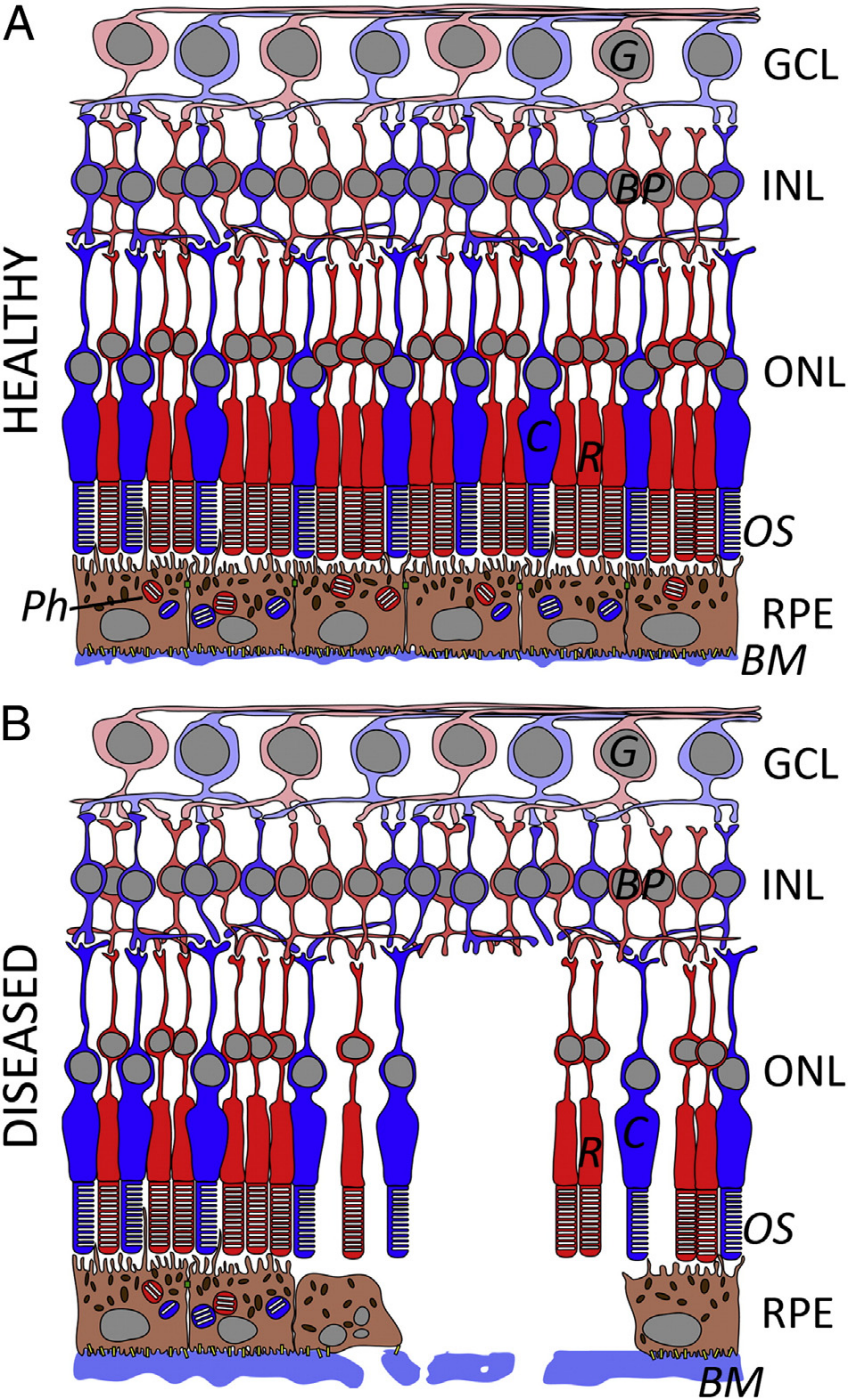
A diagrammatic comparison of healthy (A) and diseased (B) retina highlighting the strong interdependence between photoreceptors and RPE. In the diseased retina, a decreased number of cones (C) and rods (R) is associated with RPE cell loss. This may lead to RPE detachment, where the RPE is lifted off the Bruch's membrane (BM) overlying the choroid. In addition, there is a reduction of phagosomes (Ph) in the RPE, as well as decreased phagocytosis of POS. In contrast, the neural circuits comprising bipolar cells (BP) and ganglion cells (G) remain comparatively unchanged. INL inner nuclear layer; ONL outer nuclear layer; GCL = ganglion cell layer. Adapted from Ramsden *et al.*[5].

Retinal degeneration is one of the leading causes of irreversible blindness worldwide with limited effective treatment options. Retinal degeneration is the end point of many differing disease processes. In 2014, inherited retinopathies overtook diabetic related causes for blind registration in the working population in the UK [1] and in older individuals the major cause for sight loss is age related macular degeneration (AMD) [2] with over 40 million sufferers worldwide. The retina is located in the posterior chamber of the eye, lining its inner surface and comprises multiple layers of differing cell types. The majority of primary retinopathies affect the outer retina, which is primarily formed of the photoreceptors and its monolayer of support cells termed the retinal pigment epithelium (RPE) [3]. Inherited retinopathies can affect the development of the light-sensitive photoreceptor cells of the retina, the function of retinal and RPE cells, or can result in the premature loss of these cells (Fig. 1). As the intrinsic regenerative capacity of the retina and RPE is limited, one potential therapy is cellular replacement [4].

Over the past decade, stem or progenitor cell transplantation as a means of replacing tissue has evolved rapidly, with the development of protocols to drive embryonic stem cells (ESCs) towards the fate of both photoreceptors or the RPE [6] [5–15] Despite this, regenerative therapy is only in the early stages of clinical trial and is currently being developed primarily for the more common retinal degenerations and each disease poses its own challenges. Therefore, in order to learn more about these wide ranging pathologies, there is a need to develop robust models to target therapies. Until recently in retinal biology, animals have been used primarily to model disease. While this has benefits in examining an organ in relation to an organism, there are major drawbacks in terms of increasing ethical objection to the use of animals in research and critical differences between human and animal species, for example the absence of a macular region in the common rodent animal models.

In 2006, a major advance in stem cell technology produced a new means with which to investigate inherited diseases, such as retinopathies, in diseased patient cells *in vitro*. In a phenomenal series of experiments, Takahashi and Yamanaka identified the embryonic transcription factors that are required to turn an adult somatic cell into a pluripotent stem cell. The group was able to reprogram mouse [16] and subsequently human fibroblasts [17] into stem cells, termed induced pluripotent stem cells (IPSCs) using retroviral transduction with the four transcription factors OCT4, SOX2, KLF4 and c-MYC. Using this technology, fibroblast cells, which are readily accessible in the form of a skin biopsy, can be taken from sufferers of retinal diseases and converted into IPSCs. IPSC-derived eye cells will provide us with a new platform to investigate diseases in cell types, which have previously been inaccessible. In this article, we explore the current state of published outer retinal disease models using IPSCs and the technical difficulties encountered in their generation.

## 2 Technical Challenges for IPS Generation, Differentiation and Use

IPSCs can be generated from a wide array of sources including fibroblasts, keratinocytes, and T cells. The cell source can contribute to the epigenetic memory and serves as a challenge for IPSC research. Numerous studies have highlighted the varied growth and differentiation characteristics of IPSC lines. This is thought to be caused by a combination of genetic and epigenetic variation leading to subtle but significant differences in endogenous signalling. [18–21] Therefore, recent methods for deriving IPSCs from somatic cells may not always yield uniform lineage competencies between lines [40]. This necessitates optimization of IPSC-retinal cell differentiation protocols to suit each IPSC line. This fact, in combination with the long time scales and low efficiency of the majority of differentiation protocols makes for a long and expensive period of research and development before a reliable method for generating retinal cells from IPSCs can be established.

**Fig. 2:**
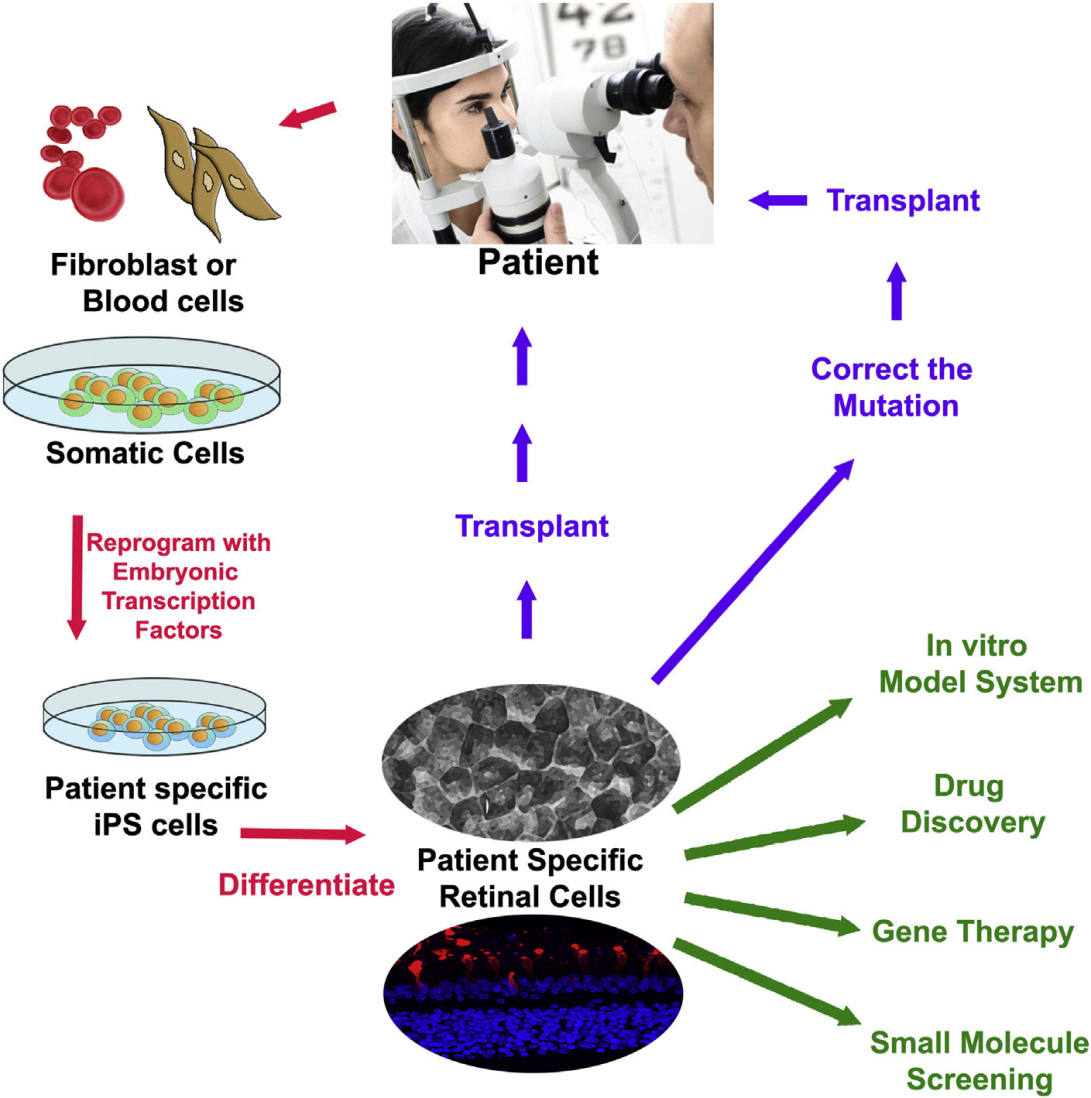
Application of patient specific IPSCs for disease modelling, drug discovery, gene therapy, small molecule screening and cell transplantation. Patient-specific IPSCs can be generated *via* genetic reprogramming of dermal fibroblasts or blood cells to pluripotency using retroviral transduction with the four transcription factors. This technology has emerged as a promising tool for identification of disease causing mutations, examining efficacy of new therapeutics, and as a cell source for autologous retinal cell replacement.

Patient IPSC-derived cells/tissues can then be used to span a range of applications from elucidating the mechanism of disease causing mutations to drug and gene therapy testing (Fig. 2). The experimental process requires that IPSC lines are also generated from healthy control individuals in order to measure differences in the mutation carrying patient cells [50]. An alternative strategy is to generate isogenic control cell lines where the mutation of interest is corrected in patient cells using gene editing technology. Control and test cell lines must then be differentiated simultaneously into the target cell. In order to make valid comparisons, it is ideal that the identity and maturity of the cells derived from both test and control cell lines are equal. Extensive studies of ESC lines has shown differences in their innate differentiation propensity [15,22]; thus it is likely that subjecting two cell lines to the same protocol could generate a different array of cell types. This could become problematic if retinal cells are identified and isolated from differentiated stem cell cultures based on the expression of markers that are also expressed in a range of neuronal cell types (*e.g.* Pax6, Chx10). Similarly, markers that are specific to a level of cell maturity are important. For example Recoverin is expressed early in human retinal development [23] and persists thereafter; therefore, using Recoverin expression as the *de facto* inclusion criteria for IPSC derived photoreceptors would include a range of cell types from developmentally immature progenitors to photoreceptors. If these potential pitfalls are not properly accounted for, incorrect conclusions about disease aetiology may be drawn.

The pure population of differentiated cells often has a limited proliferative capacity necessitating continued derivation from the original pluripotent IPSC bank [24]. IPSCs may incur mutations and chromosomal loss over time in culture as well as a secondary shortening of their telomere and reduced cell growth making the diligent maintenance of the cell bank crucial [26,27].

Thus far IPSCs have been used to generate several cell types that are implicated in retinal degenerative diseases, including RPE [28], retinal ganglion cells [29] and photoreceptors at various stages of maturity from progenitors [30] to opsin expressing, inner segment bearing, ciliated cells [31,32] reminiscent of developing photoreceptors at foetal week 12–15 of human development [23]. Three dimensional ‘optic cups’ containing multiple cell types (rod and cone progenitors, inter-neurons and ganglion cells) in a highly ordered structure have also been generated [31,32]. Despite these successes it is widely acknowledged that coaxing pluripotent cells to reliably and efficiently differentiate towards the desired retinal lineage is a considerable challenge.

Protocols for the generation of retinal cells from IPSCs employ either spontaneous or directed methods [33]. The former does not require the addition of small molecules or growth factors but simply the withdrawal of factors, which are required to maintain pluripotency from the cell maintenance media (*e.g.* basic fibroblast growth factor). While this technique has repeatedly proven to be a reliable and cost effective method for generating RPE, the production of neural retinal cell types requires a more directed procedure. Such methods commonly involve the agonism or antagonism of developmentally critical signalling pathways with small molecules or recombinant growth factors. Photoreceptor generating protocols are notoriously laborious, time consuming and highly dependent on the cell line used and epigenetic status, which can vary over time in culture [18,34,35]. As such stem cell-derived RPE is a much more easily producible, predictable and robust cell type in comparison to stem cell-derived photoreceptor-like cells.

Frequently the cell type of interest emerges alongside a myriad of contaminating cell types — being able to identify and isolate the cells of interest is critical to the success of these studies. The highly pigmented RPE can be easily identified visually and separated manually or by fluorescence activated cell sorting equipment (FACS). The isolated RPE cells then have a degree of proliferative potential over a limited number of passages, making it possible to generate sufficient material for experiments. In contrast, non-pigmented neural retinal cells require more innovative methods for their visual identification; for example ESC lines have been developed with GFP tagged expression of early eye field marker proteins to allow easy identification and purification [12].

Additionally once the desired cell type has been generated, the disease model may be hampered by the relative immaturity of the cells generated. For example studies in human ESC derived RPE have found its transcriptome to more closely match human foetal RPE than adult cells [36,37]. Where degenerative retinal diseases with a late age of onset are of interest, it will be necessary to consider whether the IPSC derived cells are a model of a pre-symptomatic stage of the disease. Innovative methods to artificially age cells in culture are being developed to counter this problem in other disease models [38].

Finally, once the desired cell type has been generated in culture, the use of this cell as a disease model may be affected by its isolation as a single cell type. *In vivo* these cells would be exposed to exogenous stresses, from a neighbouring cell type, the immune or vascular system. Exposure to these types of stresses may be required to bring out the disease phenotype in cultured cells, which may limit the utility of the IPSC ‘disease in a dish’ paradigm.

## 3 Diseases Modeled

### 3.1 Retinitis Pigmentosa (RP)

Retinitis Pigmentosa is the most common form of inherited progressive retinal dystrophy that is both clinically and genetically heterogeneous. It is characterized by the progressive loss of rod photoreceptors then RPE cells. Symptoms include night blindness and progressive visual field loss, often leading to complete blindness. No definitive treatment has been established yet. RP can be inherited in autosomal dominant, autosomal recessive, X-linked, mitochondrial and genetically more complex modes [51,52]. Up to the present time, over 60 different genes have been associated with RP, which can occur in the retina alone or together with other syndromic disorders [53]. As a result of the genetic heterogeneity, the roles of these individual causative mutations have not been fully elucidated.

IPSC-derived retinal cells are an ideal *in vitro* model for disease discovery. In effect, they enable a better appreciation of the molecular and histological basis of diseases, including, RP, but also deliver a means of identifying possible therapeutic strategies [7,54]. For example, Jin et al. [54] obtained fibroblast cells from five RP patients with distinct mutations in the *RP1*, *RP9*, *PRPH2* or *RHO* gene, and generated patient-specific IPSCs that were then differentiated into photoreceptor-like cells, which expressed characteristic immunocytochemical markers and electrophysiological properties. They found that, as occurs in disease, the number of the patient-derived photoreceptor-like cells decreased more rapidly *in vitro* than those derived from control lines. Additionally, cells derived from patients with *RP9* or *RHO* mutations expressed markers for oxidative or endoplasmic reticulum (ER) stress.

The study also looked at stem cell therapy as a useful tool for screening of drug responses in RP. The authors identified α-tocopherol as a potential therapeutic that could preserve RHO-positive cells in IPSC-derived retinal cultures carrying the *RP9* mutation. In effect, using IPSC-derived cells in drug screens may ultimately help to narrow the disease targets for experimental drugs, identify drugs for repurposing and facilitate clinical trial design. Using IPSC-derived cells as a novel platform for *in vitro* Phase 0 clinical trials would also provide important information about the efficacy and efficiency of potential therapies long before expensive clinical trials in patients. Drawbacks of this study include the use of limited photoreceptor markers and the small number of mutations analysed. In effect, the four mutations are not representative of this heterogeneous disease entity. In addition, the study does not take into account other factors that may have induced accelerated photoreceptor cell loss.

Similarly, Yoshida et al. [55] generated an IPSC line from the somatic cells of a patient with RP who carried a mutation in the *RHO* gene (E181K). This IPSC line was subsequently used to derive rod photoreceptor like cells with the same mutation. These cells were used to demonstrate that the mutation was indeed a pathogenic mutation and were used to explore the underlying molecular mechanisms and potential therapeutic approaches. With the use of an adenoviral vector gene transfer, the mutation was amended in the patients' IPSCs and the cells were then differentiated to photoreceptor-like cells. The study found a reduced survival rate in the photoreceptor cells with the E181K mutation, which was associated with a higher expression of ER stress and apoptotic markers. Furthermore, it was shown that numerous reagents (*e.g.* rapamycin, PP242, AICAR, NQDI-1 and salubrinal) promoted the survival of the patients' IPSC-derived photoreceptor-like cells, with a concurrent decrease in ER stress and apoptosis markers. This study is valuable as it draws attention to the use of IPSCs for review of disease pathophysiology, as well as for development of drug and gene therapeutics.

Furthermore, Tucker and colleagues [56] derived IPSCs from a patient with sporadic RP. Human IPSCs were used to confirm the pathogenicity of a homozygous Alu insertion into the causal gene *via* exome sequencing. In this study, the authors demonstrated that the insertion of the Alu sequence into exon 9 of the patient's *male germ cell*-*associated kinase* (*MAK*) gene blocked the generation of a splice variant of MAK, which is normally expressed only in retinal precursors. The abnormal splicing of MAK impeded normal photoreceptor development, leading to irreversible cell loss. In this study, IPSCs offered an effective means of recognizing the disease mechanism by enabling the *ex vivo* study of patient-specific retinal cells. Even though mutations in *MAK* only represent 1% of RP causes in the general population, they are quite common among individuals of Ashkenazi Jewish descent (reported in one third of cases) [57]. Nonetheless, the study acknowledges the drawbacks of using exome capture methods, especially when studying autosomal recessive diseases. In compound heterozygotes, both mutant alleles are not always recognized and other plausible disease causing heterozygous variants (*e.g.* ABCA4 and USH2A) cannot be discounted.

Schwarz et al. [58] generated IPSC derived RPE from patients with RP carrying a premature stop mutation in the *RP2* gene. RP2 protein was undetectable in the patient cells, implying that the mechanism underlying disease progression is caused by complete lack of RP2 protein. Using translational read-through inducing drugs, Schwarz et al. [58] were able to reinstate up to 20% of endogenous, full-length RP2 protein in cells carrying the mutation. This subsequently allowed the reversal of cellular phenotypic defects seen in both the affected patient fibroblasts and IPSC–RPE cells. The study successfully restored RP2 function, therefore adding to therapeutic options for a wide range of diseases caused by mutations that introduce premature stop codon into the coding sequence. However, the study only focuses on one specific mutation, therefore making it difficult to project or extrapolate results.

Li and colleagues [59] successfully used human IPSC–RPE cells as a recipient for gene therapy to replace Membrane Frizzled-Related Protein (MFRP); a gene implicated in RP that is expressed in the RPE and is thought to control actin organization with the help of CTRP5. Adeno-associated virus (AAV) 8-mediated delivery of MFRP into IPSC-derived RPE from a patient with MFRP-associated RP appeared to successfully restore actin organization observed in control cells. This gene therapy approach also showed encouraging results in mouse models. Overall, the study highlights that the successful restoration of MFRP in diseased IPSC–RPE may be a good indicator for this therapeutic approach in subsequent clinical trials. However, the study only focuses on one mutation, therefore making it difficult to extrapolate results and limitations of AAV include vector production and the limited transgene capacity of the particles [60].

### 3.2 Usher Syndrome (USH)

Usher syndrome is an autosomal recessive hereditary disorder characterized by RP and congenital sensorineural hearing loss, with a varying age of onset and extent of vestibular dysfunction. A number of causative genes have been identified including the *USH2A* gene that is expressed in the photoreceptor. Zahabi et al. [61] confirmed that several retinal-disease specific IPSC lines (including USH) can be differentiated into RPE cells, although it must be stressed that Usher's syndrome is primarily a disease of photoreceptors not RPE and so limited conclusions related to USH could be drawn from these cell lines. Tucker et al. [62] performed exome sequencing on a patient with RP and identified a probable causative mutation in *USH2A*. Sanger sequencing of the *USH2A* gene introns revealed a second mutation in intron 40. In order to further interrogate the pathophysiology, they reprogrammed the keratinocytes of the patient into IPSCs and then used direct-differentiation protocols to produce bilayered optic vesicle-like structures, comprising RPE and primitive photoreceptor like cells. cDNA analysis confirmed that the mutation in intron 40 caused expression of this intronic region, a frameshift and premature stop codon. Scrutiny of the protein expression pointed towards misfolding and potential endoplasmic reticulum stress.

### 3.3 Leber Congenital Amaurosis (LCA)

Leber congenital amaurosis is a rare degenerative inherited eye disease that can lead to severe visual impairment before the age of one [68]. It is thought to be caused by abnormal development of photoreceptor cells, or by the premature degeneration of retinal cells [68]. It is characterized by nystagmus, sluggish or no pupillary responses and poor vision [69]. Mutations in 18 different genes have been reported to cause LCA, which is an autosomal recessive disease. A third of patients carry mutations in *CEP290*, a gene which normally produces a cilium-associated protein that is involved in photoreceptor outer segment (POS) trafficking and ciliogenesis [70–73]. Treatment options remain limited [74], despite the emergence of therapeutic gene replacement [65,66,75–80].

Understanding the pathophysiology and mutation-specific disease severity in LCA patients is key to refining treatment and improving outcomes. A study by Burnight et al. [66] demonstrated a ciliogenesis defect in *CEP290*-associated LCA patient fibroblast cells. They showed that lentiviral delivery of CEP290 to patient fibroblasts increased the proportion of ciliated cells and the length of cilia in two out of three patient fibroblast lines compared to untransfected controls. This study was also able to demonstrate that the range of CEP290 therapeutic dosage was limited; with higher doses of wild-type CEP290 becoming toxic to fibroblasts cells in culture. Patient-specific IPSCs were then generated and differentiated into cells that were immunoreactive for Otx2 (expressed in almost all regions of the developing human brain) and cone opsin — although no photoreceptor morphology primitive or otherwise was demonstrated. These cells were successfully transfected with a lentiviral vectors containing full length CEP290 and the authors demonstrated expression of the full-length transcript and presence of the protein by western blot in patient derived photoreceptor precursor cells. However, the authors did not demonstrate a ciliogenesis defect in the photoreceptor precursor cells following transfection with full length CEP290, citing difficulties in identifying cilia in differentiated structures.

Several studies were able to review pathologic formation of human retinal cells *in vitro*, thereby facilitating disease modelling and drug screening. As mentioned previously, Zahabi et al. [61] generated IPSC lines from a number of patients with retinal dystrophies including LCA. A directed differentiation protocol produced cells that were pigmented and appear to be immunopositive for some RPE specific markers including MitF and RPE65. Lustremant et al. [81] used LCA patient-derived IPSCs to carry out differential transcriptome analysis in order to find genes of interest likely to be causative in the affected cell types. IPSC lines were derived from two patients with LCA carrying undetermined mutations. The IPSCs were differentiated into RPE and towards neural stem cells akin to the neural tube stage of development (not yet restricted to the eye field lineage); cell types that may be affected by the disease. Comparison of diseased cells to healthy controls identified 4 candidate genes that were differentially expressed in LCD-derived cells and could correlate with disease mechanisms associated with protein degradation and oxidative stress in this patient: *tripartite motif containing 61* (*TRIM61*) gene, the *zinc finger protein 558* (*ZNF558*) gene, *glutathione S*-*transferase theta 1* (*GSTT1*) and *neuronatin* (*NNAT*).

### 3.4 Gyrate Atrophy (GA)

Gyrate atrophy is a progressive autosomal recessive disorder that starts in childhood and induces diffuse atrophy of the choroid, RPE and sensory retina. In GA, stem cell-based disease models are useful for pharmacological screening and the evaluation of new therapeutics.

Meyer et al. [7] reported the reinstatement of ornithine aminotransferase (OAT) enzyme activity in GA IPSC-derived RPE after vitamin B6 therapy. B6 supplementation is a recognized treatment for a subset of GA patients. However, the patient in question was not predicted to benefit from B6 using data derived from testing B6 effect on the OAT enzyme in fibroblasts. This highlights how important tissue specific drug testing is and showcases how powerful IPSC technology can be in non-regenerating tissues.

### 3.5 Juvenile Neuronal Ceroid Lipofuscinosis (NCL)

Juvenile NCL, also known as Batten disease is a group of severe neurodegenerative diseases characterized by intracellular accumulation of autofluorescent wax-like lipid pigments (ceroid-lipofuscin) in neurons. There are several subtypes based on mutations of the various genes, disease onset, and severity of the neurological defects such as progressive dementia, seizures and visual failure. The latter is normally central in nature, due to degeneration of cones in the macula. Disease progression is very aggressive and patients often die during their second or third decade. It is inherited as an autosomal recessive genetic disorder and often caused by a genomic DNA deletion in the gene, *ceroid lipofuscinosis 3* (*CLN3*) [82]. There is currently no cure for this disorder and the molecular mechanisms have not been fully elucidated [83].

Stem cell technology is a powerful tool for analysing the pathophysiology and for enhancing drug screening. A recent study by Lojewski et al. [84] generated IPSC lines from patient with both late infantile NCL (*CLN2* mutation) and juvenile NCL (*CLN3* mutation). Patient IPSCs were differentiated into neuronal (not retinal) tissue. Abnormalities in endosomal-lysosomal system were detectable in the patient IPSC, but disease-subtype specific lysosomal storage was only evident in their differentiated neuronal derivatives. This clearly shows the necessity of IPSCs and IPSC differentiation technology to effectively model these diseases.

The abnormalities could be corrected in patient cells by using adenovirus vector to overexpress wild type protein encoded by the *CLN2* or *CLN3* gene, confirming previous outcomes in animal models [85] and lending support to AAV mediated gene therapy currently in clinical testing (www.clinicaltrials.gov, NCT01161576). These IPSC-derived neural progenitor cells were also used to screen potential pharmacological modulators of the CLN2 encoded protein, demonstrating the use of patient-derived IPSCs as a platform for testing new therapeutic candidates.

### 3.6 Best Vitelliform Macular Dystrophy (BVMD)

Best vitellliform macular dystrophy is an autosomal dominant hereditary maculopathy with childhood-onset accumulation of liposfuscin in RPE. Affected individuals develop progressive central acuity loss, and metamorphopsia, due to mutations in bestrophin, a chloride channel [86]. There are different stages in the disease process including choroidal neovascular membranes due to vitelliform lesions and geographic atrophy in later stages [87,88].

Singh et al. [89] used human IPSCs to generate RPE from BVMD patients and unaffected siblings in order to study the cellular and molecular processes underlying this disease. Several differences were observed between BVMD and normal sibling IPSC–RPE. The authors noted that IPSC–RPE from patients had disrupted fluid flux, a build-up of autofluorescent material and oxidative stress, a delay in rhodopsin degradation and differences in stimulated calcium responses following POS feeding. This study thus suggests a role for intracellular calcium regulation and oxidative stress in the disease mechanism. These important and RPE-specific functional differences would have been impossible to determine without access to actual patient RPE cells or the use of patient-derived IPSC–RPE.

### 3.7 Age Related Macular Degeration (AMD)

AMD is a complex disease that can be subdivided into a “dry” and “wet” type. It involves loss of the RPE/photoreceptor layers, thinning of the outer plexiform layer, thickening of Bruch's membrane and atrophy of the choriocapillaris. In dry AMD, there is an accumulation of lipid-like deposits (drusen) between the RPE and Bruch's membrane, occurring typically in individuals over 50. Geographic atrophy is more prominent in later stages, leading to photoreceptor cell loss. In wet AMD, the choroidal vasculature can grow in the subretinal space, termed “choroidal neovascularisation.” In view of the pathophysiology, cell replacement therapy for retinal cell loss is very appealing, with several clinical trials underway.

Generating a disease model for AMD may be useful in understanding its pathogenesis and in the development of effective therapeutic strategies. AMD has a strong genetic characteristic with over 50 different loci identified so far. These include two on chromosomes 1 and 10 leading to a high-risk haplotype. The mutation associated with chromosome 1 affects the *complement factor H* gene and subsequently the complement cascade. However, the mechanism by which the chromosome 10q locus leads to increased AMD risk remains unknown. Yang et al. [90] used an unbiased proteome screen of patient specific IPSC-derived RPE cells from patients with a high and low risk haplotype of 10q loci. By artificially aging the cells with A2E (one of the lipofuscin fluorophores that accumulate in RPE cells with age), they noted a reduced superoxide dismutase 2-mediated antioxidant defence in the high-risk haplotype. This reduced protective response to A2E may cause increased levels of oxidative stress in the RPE of affected patients. The study highlights several underlying pathogenic mechanisms, opening the doors to potential treatment options. Chang et al. [91] generated IPSCs from T cells of patients with dry AMD using integration free episomal vectors and differentiated these cells into RPE using a directed differentiation method. The AMD patient-derived RPE cells were found to have reduced antioxidant capability compared with control cells. The group then used the IPSC-derived RPE to screen a panel of dietary supplements that have previously been shown to have potential retinal protective or antioxidant capabilities. Of these compounds, treatment with 10 μm curcumin was shown to have a significant effect on cell viability. Pre-treatment with curcumin protected these AMD-related RPE cells from H_2_O_2_-induced cell apoptosis and upregulated the expression of several oxidative stress-regulating genes. Although the systemic effects of such treatments must still be evaluated in suitable animal models, this study demonstrates the potential of IPSC-derived cells to screen for the activity of therapeutic candidates in the desired target cell type early in the drug development process.

## 4 Conclusion

Stem cell technology is coming of age as a tool to model retinal degeneration; IPSCs have already been used to recapitulate normal *versus* abnormal retinal cell behaviour *in vitro* and to gain new mechanistic insights into disease aetiology. Patient specific IPSCs may be used in pharmaceutical screening and in the elucidation of new treatment options for retinal diseases (Table 1). The potential benefits in terms of reducing the attrition rate for drug candidates in early-stage clinical trials and decreasing the requirement for animal models are an obvious attraction. Many emerging developments in the field of stem cell technology offer an exceptional opportunity to treat inherited retinal degenerative diseases and finally improve patients' lives. However, there exist some obstacles with the use of IPSCs, which need to be overcome before clinical application.

## Funding

This work was supported by funding from The London Project to Cure Blindness; the Medical Research Council (MRC) UK (G1000730); the California Institute of Regenerative Medicine (CIRM) (G1000730); Fight for Sight UK (1755); The Lincy Foundation (P12761); the Macular Society; and the National Institute for Health Research (NIHR) (BRC2_011) Biomedical Research Centre based at Moorfields Eye Hospital National Health Service (NHS) Foundation Trust and University College London Institute of Ophthalmology. The views expressed are those of the author(s) and not necessarily those of the NHS, the NIHR or the Department of Health.

## Competing Interest Statement

The authors declare no competing financial interests.

## Figures and Tables

**Table 1:** Summary of disease causing mutations that have been identified and examined using IPSCs, and whether these disease models have led to drug discovery, gene introduction and discovery.

Disease	Gene	Mutation	Drug discovery	Gene introduction	Gene discovery	Reference
RP	*RP2*	R120X	✔			[58]
	*RHO*	G188R				[54]
	*RHO*	E181K	✔	✔		[55]
	*RP9*	H137L	✔			[54]
	*RP1*	721Lfs722X				[54]
	*RDS*/*PRPH2*	W316G				[54]
	*MFRP*			✔		[59]
Sporadic RP	*MAK*				✔	[56]
Usher	*USH2A*				✔	[62]
LCA	*GUCY2D*				✔	[81]
	*CEP290*		✔	✔		[66]
Gyrate atrophy	*OAT*	A226V	✔	✔		[7]
Best	*BEST1*	A146K, N296H				[89]
Juvenile NCL	*CLN2*		✔	✔		[84]
AMD			✔			[90]
